# Bone mineral density assessment according to Italian essential assistance levels: a positive predictive value analysis based on administrative data

**DOI:** 10.3389/fendo.2025.1689549

**Published:** 2025-12-12

**Authors:** Veronica Abate, Anita Vergatti, Guido Cavati, Simone Magelli, Alfonso Varriale, Daniela Merlotti, Gianpaolo De Filippo, Gaetano Piccinocchi, Lanfranco D’Elia, Luigi Gennari, Domenico Rendina

**Affiliations:** 1 Department of Clinical medicine and surgery, Federico II University, Naples, Italy; 2 Department of Medicine, Surgery, and Neurosciences, University of Siena, Siena, Italy; 3 Service de Médecine Interne et Rhumatologie, Service de Santé des Armées, Hôpital National d’Instruction des Armées Percy, Clamart, France; 4 “COoperativa sociale di MEdici di medicina GENerale A.R.L (COMEGEN)“ Medical Cooperative, Naples, Italy

**Keywords:** osteoporosis, DXA, bone mineral density, essential assistance levels, male osteoporosis

## Abstract

**Background:**

Osteoporosis (OP) is the gradual loss of bone mineral density (BMD), predisposing to fragility fractures. It can be the result of aging or menopause in primary OP or other diseases and medications in secondary OP. The gold standard diagnostic tool for low BMD is represented by dual-energy X-ray absorptiometry (DXA), which has been included among the Essential Assistance Levels (EALs) in Italy for adequate prescription. This observational study based on administrative data evaluates whether the EALs are designed to predict OP (designed to include OP screening), with subsequent BMD-DXA evaluation in Italian men and women. The SIMON database was used to select women and men scheduled to undergo BMD-DXA according to EALs.

**Results:**

BMD was evaluated in 18,087 subjects [Male (M): Female (F) 889: 17198; mean age 71.3 ± 9.3 years; body mass index (BMI): 26.9 ± 4.5 Kg/m^2^] for primary OP and in 6,503 subjects (M: F 649: 5854; mean age 70.5 ± 9.4 years; BMI: 25.4 ± 4.8 Kg/m^2^) for secondary OP, both suspected on the basis of EALs. Primary OP was confirmed by DXA in 13,759 (80.0%; mean age 71.9 ± 9.2 years; BMI: 26.5 ± 4.8 Kg/m^2^) women and in 551 men (62.0%; mean age 69.3 ± 9.4 years; BMI: 26.5 ± 4.8 Kg/m^2^). Secondary OP was confirmed by DXA in 4,683 women (80.0%; mean age 70.7 ± 8.9 years; BMI: 25.3 ± 5.0 Kg/m^2^) and in 514 men (79.2%; mean age 69.7 ± 9.4 years; BMI: 26.3 ± 5.3 Kg/m^2^). The positive predictive values of EALs for OP were higher among women for primary OP than men and similar in both genders without any significant difference in secondary OP.

**Conclusion:**

Italian EAL are a useful diagnostic tool for primary of OP in both sexes, with significant gender dimorphism evident in secondary OP.

## Introduction

Osteoporosis (OP) is the most common metabolic bone disorder, characterized by a silent reduction of bone mineral density (BMD) and microarchitectural damage, predisposing to fragility fractures. Primary OP develops as a result of aging or menopause-related bone demineralization, while secondary OP is the consequence of pathological conditions or medications that reduce bone mass and increase fracture risk (1). OP affects 6.3% of men over 50 years of age and 21.2% of postmenopausal women worldwide (2), causing about 9 million fractures annually (3). Fragility fractures worsen the quality of life, causing disability and increasing morbidity and mortality. Moreover, it leads to a significant impact on national health care systems, with costs ranging between 5000 and 6500 billion USD annually in Canada, Europe, and the USA just for hospitalization and surgery (4). Given the progressive aging of the population, both are expected to increase by 2050 by 310% and 240% in men and women, respectively, according to estimates (4, 5). In Italy, the economic burden is equal to 6% of the Italian healthcare expenditure, exceeding the European average of between 2.5% and 3.5% (6). In this setting, the prevention of fragility fractures (both primary and secondary) represents one of the best strategies to reduce social and financial costs. However, the prevalence of OP appears to be different between men and women, with higher values for the latter. On the other hand, men showed a shorter life expectancy during the first six months after hip fractures and a higher mortality compared to women (7).

The actual gold standard method for the evaluation of bone health and mineralization is the measurement of BMD using the dual-energy X-ray absorptiometry (DXA). Several studies have confirmed that low BMD predicts fragility fractures (8–10). Considering the significant human and economic impact, the Italian Ministry of Health provides for national funds for OP prevention within the essential assistance levels (EALs). These lasts are represented by the basic national health benefits package, which must be uniformly provided throughout the country (11). To prevent OP and fragility fractures, EALs establish the criteria for community-dwelling individuals of both sexes at increased OP risk to undergo BMD measurement by DXA. According to Italian EALs, these criteria are: (i) men and women of any age with major OP risk factors (previous fragility fractures; radiological evidence of OP; chronic therapies increasing the risk of OP; diagnosis of diseases with higher risk of OP), (ii) post-menopausal women with major risk factors (family history of bone fracture at age < 75 years; body mass index < 19 kg/m^2^; menopause at age < 45 years), (iii) postmenopausal women and men ≥ 60 years in the presence of at least three or more minor risk factors for OP (age > 65 years, family history of severe OP; amenorrhea > 6 months in premenopausal age; inadequate calcium intake; vitamin D deficiency; smoking > 20 cigarettes/day; alcohol abuse [> 60 g/day]) (12). We conceived this observational study based on administrative data to evaluate whether EALs are able to predict OP, confirmed by the BMD-DXA, in Italian men and women with suspected primary and secondary OP.

## Materials and methods

This study is a cross-sectional diagnostic yield study using routinely collected data from the SIMON (*SIndrome Metabolica Osteoporosi e Nefrolitiasi*, Metabolic Syndrome, Osteoporosis, and Nephrolithiasis) database and part of the SIMON protocol (13–17). Briefly, general practitioners of the “COMEGEN” (*COoperativa di MEdicina GENerale*) Medical Cooperative, operating within one of Naples Local Health Care Units (*Azienda Sanitaria Locale, ASL Napoli 1*), selected all the patients scheduled to undergo, from December 2001 to June 2018, a BMD evaluation by DXA [International Classification of Diseases–9th revision (ICD9) code 8898], according to Italian EALs (12). Data was extracted on 1st June 2018. The endpoint of this study was the assessment of the diagnostic yield by considering positive predictive values of EALs among EAL-referred patients.

### Inclusion criteria

All subjects aged more than 40 years old, with eligibility for a BMD-DXA scan according to EALs, were included in the study.

### Exclusion criteria

**Figure 1:**
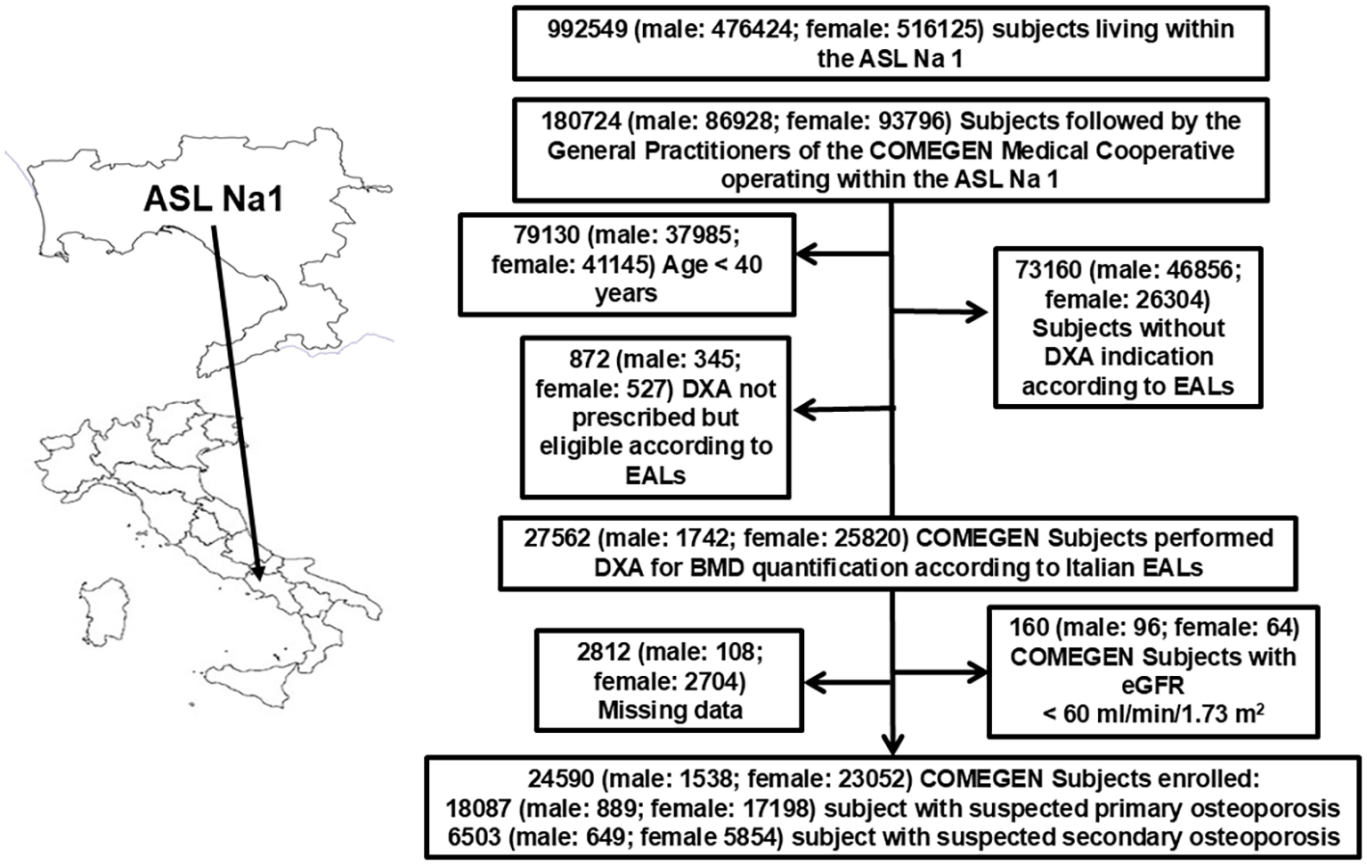
Study flowchart of selected subjects followed by COMEGEN general practitioners.

Age lower than 40 years; DXA not prescribed but patient eligible according to EALs; estimated glomerular filtration rate < 60 ml/min/1.73 m^2^ [eGFR, ICD9 codes 5853 to 5859, 586, and 6393 (18)] and missing data (**Figure 1**).

### OP diagnosis

OP’s diagnosis was based on DXA results using the female reference database for T-score of all ethnic groups (T-score ≤ −2.5 SD at any site), according to World Health Organization (WHO) criteria (19), and/or on medical history of fragility fractures (hip, vertebral, wrist, and/or humerus) according to WHO criteria (20) and to the International Osteoporosis Foundation (21), and to the International Society of Clinical Densitometry (ISCD) (22). In case patients performed more than one DXA scan evaluation, just the first one was considered for the scope of the study. OP patients were divided into primary and secondary OP according to ICD9 codes and according to the latest Italian osteoporosis and mineral metabolism diseases society (Società Italiana Osteoporosi e Malattie del Metabolismo Minerale, SIOMMMS) guidelines for diagnosis, prevention, and treatment of OP (23).

### Secondary OP diagnosis

The diagnosis of secondary OP was made for patients with malabsorption syndromes (ICD9 codes 5793–5799), rheumatoid arthritis (ICD9 code 7140), long-term immobilization, hyperthyroidism (ICD9 codes 24200 to 24291), primary hyperparathyroidism (ICD9 codes 25200–25208), hypoparathyroidism (ICD9 code 2521), Cushing’s syndrome (ICD9 code 2550), chronic liver disease (ICD9 codes 5710–5719), pituitary tumors (ICD9 codes 1943, 2273, 2370), surgical history of terminal ileal resection (ICD9 code 4562), gastrectomy or small bowel bypass (ICD9 codes 430 to 4499), eating disorders (ICD9 codes 3071 and 30750–30759), alcoholism (ICD9 codes 30390–30393), regular use of gonadotropin-releasing hormone agonist, glucocorticoids (more than 5 mg/day of prednisone or equivalents for more than 3 months), anticonvulsants, heparin, cytotoxic agents for a current or past diagnosis of cancer and cigarette smoking > 20 cigarettes/day. In subjects without any cause of secondary OP, the diagnosis of primary OP was performed by exclusion.

The SIMON study was approved by the ASL Naples 1 Ethics Committee (protocol number 0018508/2018).

### Data collected

We collected data regarding age, gender, body mass index (BMI, measured by general practitioners), eGFR (18), all pharmacological treatments, DXA results, and EALs criteria for the DXA exam.

### Evaluation of consistency of the results

To validate the administrative data obtained, the ICD9 codes were matched with medical records from the COMEGEN dataset. A group of subjects randomly selected from a pre-generated list of females and a list of males. A population of 24,590 (1,538 males and 23,052 females) was assumed to calculate a sample (5% margin of error and 90% confidence levels).

### Statistical analysis

The statistical analyses were performed using the IBM SPSS Statistics software, version 28 (International Business Machines Corporation, Armonk, New York). The data distribution was assessed by the Kolmogorov-Smirnov test, and all variables had a normal distribution. Student’s t-test, Pearson’s chi-squared test, and chi-squared test for proportion were used for statistical comparisons for continuous variables and dichotomous variables, respectively. Positive predictive values (PPV) of EALs were calculated as percentages by dividing the number of true positive subjects (affected by OP according to DXA and EALs positive for OP) by the number of subjects suspected of having OP according to EALs, that is, true positive + false positive (subjects who performed BMD by DXA and resulted positive to DXA and EALs + subjects who resulted negative for DXA but positive for EALs, respectively). In summary, for the purpose of the study, the PPV indicates high reliability of the EALs to predict the diagnosis of OP that would be eventually confirmed by a BMD evaluation by DXA. A *p*-value < 0.05 was considered significant. The results are reported as mean (standard deviation – SD), or absolute, or percentages.

## Results

On 1st June 2018, among the 180724 subjects followed by COMEGEN general practitioners, 28434 subjects (M: F 2087:26347; mean age 71.2 ± 8.8 years; BMI: 26.5 ± 4.6 Kg/m^2^) were eligible for BMD evaluation according to EAL criteria. In 16.5% of males and 2.0% of females, the DXA examination was not prescribed, even if the patient was eligible according to EALs, as in **Figure 1**.

After the application of all exclusion criteria, the population counted 24,590 subjects (**Table 1**) in whom DXA was prescribed according to EALs criteria (15), as shown in **Figure 1**. Among them, 18,087 subjects (M: F 889: 17198; mean age 71.3 ± 9.3 years; BMI: 26.9 ± 4.5 Kg/m^2^) were evaluated because of suspected primary OP, and 6,503 subjects (M: F 649: 5854; mean age 70.5 ± 9.4 years; BMI: 25.4 ± 4.8 Kg/m^2^) for suspected secondary OP.

**Table 1:** Clinical and biochemical characteristics of the study population classified according to gender.

	Male	Female
N (*n*; %)	1538; 6.3	23052; 93.7
Age (years)	70.9 ± 8.7	71.1 ± 8.9
BMI (Kg/m^2^)	26.7 ± 4.8	26.4 ± 4.9

n, number; BMI, body mass index (measured by general practitioners).

Of the 18,087 subjects who underwent DXA for primary OP, the diagnosis was confirmed in 14,310 of them (79.1%; M: F 551: 13759; mean age 71.8 ± 9.4 years; BMI: 26.5 ± 4.6 Kg/m^2^). Analyzing the data by gender, the diagnosis was confirmed in 13,759 (mean age 71.9 ± 9.2 years; BMI: 26.5 ± 4.8 Kg/m^2^) out of 17,198 women. The PPV of EAL’s criteria for the diagnosis of primary OP confirmed by DXA in women was 80.0%. In men, the diagnosis of primary OP was confirmed in 551 (mean age 69.3 ± 9.4 years; BMI: 26.5 ± 4.8 Kg/m^2^) out of 889. The PPV of EAL’s criteria for the diagnosis of primary OP confirmed by DXA in men was 62.0%. As reported in **Table 2**, the prevalence of confirmed diagnosis of primary OP was significantly higher in women than in men (*p* < 0.01). EALs showed higher PPV in women for primary OP. A comparison between genders was also performed to obtain a difference of 18.0% (95% C.I. = 13.9 to 22.11) in PVV (χ^2^ = 103.40; *p* .< 0.0001).

**Table 2:** Prevalence of osteoporosis diagnosis in males and females by Dual-X-Energy Absorptiometry for suspected primary and secondary osteoporosis according to Italian Essential Assistance Levels.

	Osteoporosis diagnosis		p
	Confirmed	Not-confirmed	
Primary Osteoporosis			
- Males	551 (62.0)	338 (38.0)	< 0.01
- Females	13759 (80.0)	3439 (20.0)
Secondary Osteoporosis			
- Males	514 (79.2)	135 (21.8)	0.71
- Females	4683 (80.0)	1171 (20)

Data are expressed as absolute number (percentage). The osteoporosis diagnosis was based on Dual-X-Energy Absorptiometry results (10.1002/jbmr.5650090802), and/or to medical history of anti-osteoporotic treatment (https://www.aifa.gov.it/documents/20142/1728074/nota-79.pdf.), and/or medical history of fragility fractures (https://www.who.int/news-room/fact-sheets/detail/fragility-fractures<ns/>:~:text=Fragility%20fractures%20result%20from%20low,in%20the%20absence%20of%20osteoporosis; https://www.osteoporosis.foundation/health-professionals/fragility-fractures<ns/>:~:text=Fragility%20fractures%2C%20which%20result%20from,2019%5D). ICD9: International Classification of Diseases–9th revision. Secondary osteoporosis was diagnosed in patients with malabsorption syndromes (ICD9 codes 5793–5799), rheumatoid arthritis (ICD9 code 7140), long-term immobilization, hyperthyroidism (ICD9 codes 24200 to 24291), primary hyperparathyroidism (ICD9 codes 25200–25208), hypoparathyroidism (ICD9 code 2521), Cushing’s syndrome (ICD9 code 2550), chronic liver disease (ICD9 codes 5710–5719), pituitary tumours (ICD9 codes 1943, 2273, 2370), surgical history of terminal ileal resection (ICD9 code 4562), gastrectomy or small bowel bypass (ICD9 codes 430 to 4499), eating disorders (ICD9 codes 3071 and 30750–30759), alcoholism (ICD9 codes 30390–30393), regular use of gonadotropin-releasing hormone agonist, glucocorticoids, anticonvulsants, heparin, vitamin A, cytotoxic agents for a current or past diagnosis of cancer and cigarette smoking > 20 cigarettes/day. The primary osteoporosis was diagnosed in subjects without any cause of secondary osteoporosis.

Among the 6,503 subjects examined by DXA for secondary OP, the diagnosis was confirmed in 5,197 of them (79.9%; mean age 70.6 ± 9.1 years; BMI: 25.4 ± 4.9 Kg/m2). Analyzing the data by gender, the diagnosis of secondary OP was confirmed in 4,683 (mean age 70.7 ± 8.9 years; BMI: 25.3 ± 5.0 Kg/m^2^) out of 5,854 women. The PPV of EAL’s criteria for the diagnosis of secondary OP confirmed by DXA in women was 80.0%. On the other hand, the diagnosis was confirmed in 514 (mean age 69.7 ± 9.4 years; BMI: 26.3 ± 5.3 Kg/m^2^) out of 649 men. The PPV of EAL’s criteria for the diagnosis of secondary OP confirmed by DXA in men was 79.2%. As reported in **Table 2**, the prevalence of secondary OP for diagnosis of secondary OP was not significantly different between women and men (*p* > 0.05). The comparison between males and females showed a difference of 0.80% (95% C.I. = −2.89 to 4.49) in PVV (χ^2^ = 0.14; *p* < 0.71).

Given the disagreement on smoke and alcohol abuse as causes of secondary OP (23), subjects with minor risk factors, such as cigarette smoking > 20 cigarettes/day and alcohol intake, were excluded from the secondary OP group. The secondary OP group from the sub-analysis was composed of 3008 subjects. The diagnosis was confirmed in 2,445 of them (81.3%; mean age 70.5 ± 7.8 years; BMI: 25.2 ± 4.8 Kg/m^2^). Data by gender confirmed the diagnosis of secondary OP was confirmed in 2,277 (mean age 70.4 ± 8.2 years; BMI: 25.3 ± 4.9 Kg/m^2^) out of 2,797 women. The PPV of EAL’s criteria for the diagnosis of secondary OP in this subgroup was 81.4%. The diagnosis was also confirmed in 168 (mean age 70.7 ± 7.6 years; BMI: 25.6 ± 4.8 Kg/m^2^) out of 211 men, 80.0%.

### Consistency of the results

A group of 686 subjects (male 308; 44.9%, female 378; 55.1%; mean age 69.8 ± 7.9 years; BMI: 25.9 ± 4.4 Kg/m^2^) was selected to obtain a 5% margin of error and 90% confidence level. The ICD-9 matched with the diagnosis posed in the medical records.

## Discussion

Our study results firstly demonstrate that in a population of 24,590 subjects screened for EALs criteria, a very small percentage of subjects were represented by men, despite EALs setting 60 years of age as a benchmark to examine BMD by DXA in men. These results indicate that EALs lower uptake among men in this cohort and suggest the need to increase awareness in this population group, given their high risk for fragility fractures (24). On the other hand, EALs’ criteria to prescribe DXA examination had different PPVs for primary OP diagnosis in men and women (62.0% in men and 80.0% in women), performing better in women. For secondary OP, PPVs for both genders were similar and without significant differences, even in a subgroup of subjects not considering alcohol and cigarette smoking as risk factors. According to our results, EALs appear adequate to detect patients with secondary OP in both genders, showing similar results in terms of PPV. As to primary OP, the PPVs branch off in men and women, showing good reliability for women but not men, and failing to properly identify the latter. Consequently, according to our data, if EALs were to be fully applied, 20% of men at low risk of having primary OP would undergo DXA, resulting in unnecessary exposure to radiation and unnecessary economic burden on the health care system.

OP and fragility fractures are worldwide issues of significant social and economic impact, for which timely and accurate diagnosis is crucial for effective patient management and public health control. To achieve this, several institutions, such as the World Health Organization, have developed and implemented strategies for early detection of OP, indicating DXA as the gold standard method for BMD quantification (25). However, as it exposes patients to very low, albeit significant, doses of ionizing radiation, some clinical recommendations need to be taken into account for its prescription (26). Several Preventive Task Forces, indeed, suggest against screening in men or found insufficient results to take a stand. Among them, the Canadian Task Force on Preventive Health Care recommends against screening men and younger women, suggesting good clinical practice to identify patients at high risk of fragility fractures (27). On the other hand, the U.S. Preventive Service Task Force also concludes that the current evidence is insufficient to assess the balance of benefits and harms of screening for OP to prevent osteoporotic fractures in men (28). In Italy, clinical recommendations for the prescription of DXA are clearly described by EAL’s criteria. A high positive predictive value would suit such a condition for the early detection of OP before the occurrence of fragility fractures and to reduce the chances of missing a diagnosis.

Latest guidelines for male OP indicate a threshold for BMD and suggest risk assessment by a computer-based algorithm, such as the FRAX score, to initiate a treatment (29). However, they fail to indicate criteria to firstly request BMD evaluation by DXA in the male population. At the same time, as above, a whole population screening is not recommended (27). Therefore, it seems necessary to establish international criteria to screen patients at risk of fragility fractures before the BMD evaluation to avoid underestimation and unnecessary examination at the same time.

Ultimately, the gold standard approach through DXA represents the best interpretation of the results. It is indeed possible that EAL’s criteria screen men and women appropriately, but if for women the BMD evaluation confirms the suspected diagnosis, it fails for men. Appropriate and more in-depth studies are needed to clarify whatever option is valid in men.

It must also be noted that one of the add-on issues brought by the study is the substantial disparity existing in the screening cohort and in the pattern of care provided to men compared to women, with the male sample size (*n* = 1,538) being significantly smaller than the female sample size (*n* = 23,052). This observation reflects the same results collected around the world and boosts the need to raise awareness in men (30). However, it must be considered that the gender disparity in the population limits the generalizability of the results.

This study presents strengths and limitations. Its strength consists in the high number of individuals enrolled, which ensures a high generalizability of the results. The worldwide generalizability of its results is also guaranteed by the overlap between EAL’s criteria and ISCD indications for DXA prescription. Moreover, the absence of a defined screening plan for male OP is defined upon evidence (31), as for Canadian and U.S. Preventive Task Force, anticipate results on DXA recommendation in men according to Italian criteria. This study on the Italian experience lays the foundation for further studies to define criteria to prescribe DXA in men. The study also presents its limitations. Indeed, the use of ICD codes represents both a strength and a limitation: ICD9 classification was validated in a subgroup of subjects and presents adequate accuracy for chronic conditions (32), but also a risk of misclassification. On the other hand, a study based on administrative data has other limitations, namely the lack of detailed clinical information and of centralized instrumental examinations. Indeed, all the DXAs were carried out in different clinical centers, even if affiliated with the Italian Health National System (in Italian Sistema Sanitario Nazionale) and therefore subjected to quality controls (International Organization for Standardization—ISO 9001) (13). It should also be considered that this study was unable to provide any data about subjects not eligible for DXA according to EALs (false negative), thereby missing their information in the calculation of sensitivity, specificity, and negative predictive values of EALs for the early detection of patients at risk of having OP. Lastly, the lack of cost or risk-benefit data represents another limitation for this study.

In conclusion, the results of our study indicate that Italian and international criteria used to prescribe BMD evaluation through DXA have a different PPV for primary OP in men and women, leading to consideration of an adjustment of the same according to gender, with specific regard for men. Further studies are needed to validate and to confirm our results in a different setting before recommending policy change. These finding warrants discussion on the potential need for gender-specific adjustments to the screening criteria.

## Data Availability

The raw data supporting the conclusions of this article will be made available by the authors, without undue reservation.
